# Case report: Neoadjuvant-intent pembrolizumab resulted in complete response in a xeroderma pigmentosum patient with locally advanced resectable cutaneous squamous cell carcinoma of the nose

**DOI:** 10.3389/fmed.2024.1488400

**Published:** 2024-10-11

**Authors:** Nader G. Zalaquett, Lara Kreidieh, Bassem Youssef, Marc Mourad, Firas Kreidieh

**Affiliations:** ^1^Faculty of Medicine, American University of Beirut, Beirut, Lebanon; ^2^Department of Otorhinolaryngology-Head and Neck Surgery, American University of Beirut Medical Center, Beirut, Lebanon; ^3^Division of Hematology-Oncology, Department of Internal Medicine, American University of Beirut Medical Center, Beirut, Lebanon; ^4^Department of Radiation Oncology, American University of Beirut Medical Center, Beirut, Lebanon

**Keywords:** anti-PD1, pembrolizumab, cutaneous squamous cell carcinoma, cSCC, xeroderma pigmentosa

## Abstract

**Background:**

Anti-PD1 antibodies have gained popularity in the treatment of skin cancers. These drugs have been FDA approved for treatment of cutaneous melanoma and unresectable/metastatic squamous cell carcinoma of the skin. However, the use of anti-PD1 antibodies is not established for resectable cutaneous squamous cell carcinoma, as the mainstay treatment is surgical excision.

**Case:**

A 49-year-old female with Xeroderma Pigmentosum presented with an ulcerating lateral nasal mass causing obstruction. Biopsy confirmed cutaneous squamous cell carcinoma and was staged as IVA (T2N2cM0) based on PET-CT findings, which showed a 2.7 × 2.3 cm left nasal mass and radiotracer-avid cervical lymph nodes. Despite surgical recommendations, the patient declined surgery due the expected morbidity and disfigurement. Instead, she received neoadjuvant Pembrolizumab (200 mg IV every 3 weeks). After two cycles, PET-CT and MRI showed significant reduction in the nasal mass and decreased cervical lymph node involvement. On physical exam, the nasal lesion had resolved. Multidisciplinary tumor board discussion recommended radiation therapy instead of neck dissection, considering the patient's clinical response and potential surgical morbidity. After a third Pembrolizumab cycle, she received 66 Gy in 33 fractions, followed by continued adjuvant immunotherapy.

## Introduction

Cutaneous squamous cell carcinoma (cSCC) is the second most common non-melanoma skin cancer after basal cell carcinoma accounting for 20% of skin cancers (1). With 1 million new cases in the United States each year, it results in up to 9,000 estimated deaths annually (1–5). Importantly, cSCC incidence is increasing across the globe. For instance, the lifetime risk for development of this cancer is estimated at 4% to 9% for women and 9% to 14% for men in the United States (6). Currently, the incidence ranges from 5 to 499 per 100,000 patients depending on the latitude (1). In addition to morbidity, cSCC translates into a great cost burden on the health system. For example, a recent study in the UK estimated an annual cost of £33 to £46 million for diagnosis and treatment (7, 8).

Several therapies are being investigated for the treatment of cSCC, including laser, photodynamic therapy, topical therapy, curettage, and cryosurgery (9). However, the mainstay and widely approved treatment for cSCC is surgical excision and adjuvant radiation therapy. This conventional approach can carry an added morbidity when lesions are located in the head and neck region with increased anatomical intricacy and potential proximity to major vessels (10).

Historically, advanced and recurrent/metastatic cSCC of the head and neck (cSCC-HN) were treated with chemotherapy in addition to radiation therapy (11). Over the past decade, the advent of immune checkpoint inhibitors (ICIs), particularly PD-1 inhibitors, such as cemiplimab, pembrolizumab, and nivolumab, has led to significant improvement in cSCC outcomes. While ICIs gained (FDA) approval following their demonstrated efficacy and acceptable safety profile, their use remained limited to patients with unresectable, metastatic, or recurrent cSCC (12). Interestingly, recent data on neoadjuvant immunotherapy has resulted in a paradigm shift in the management of cutaneous melanoma (13, 14). However, evidence regarding the role of neoadjuvant immunotherapy for resectable cSCC remains limited to date. Importantly, experience and knowledge on the use of immunotherapy for patients with rare diseases, such as Xeroderma Pigmentosum (XP), remains limited despite high predisposition to develop skin cancers among this patient population.

In this article, we report the case of a 48-year-old female with XP presenting with resectable lateral nasal cSCC. Due to expected disfigurement and fear from surgical morbidity, the patient completely refused surgical excision even after multiple attempts to convince her that surgery might be inevitable. In respect of the patient's autonomy, the medical team explored other options and started the patient on pembrolizumab monotherapy with neoadjuvant intent, despite the lack of solid evidence on its efficacy in this setting. Surprisingly, the tumor disappeared completely exhibiting a complete clinical and radiologic response, and surgical excision was no longer warranted.

## Case

**Figure 1:**
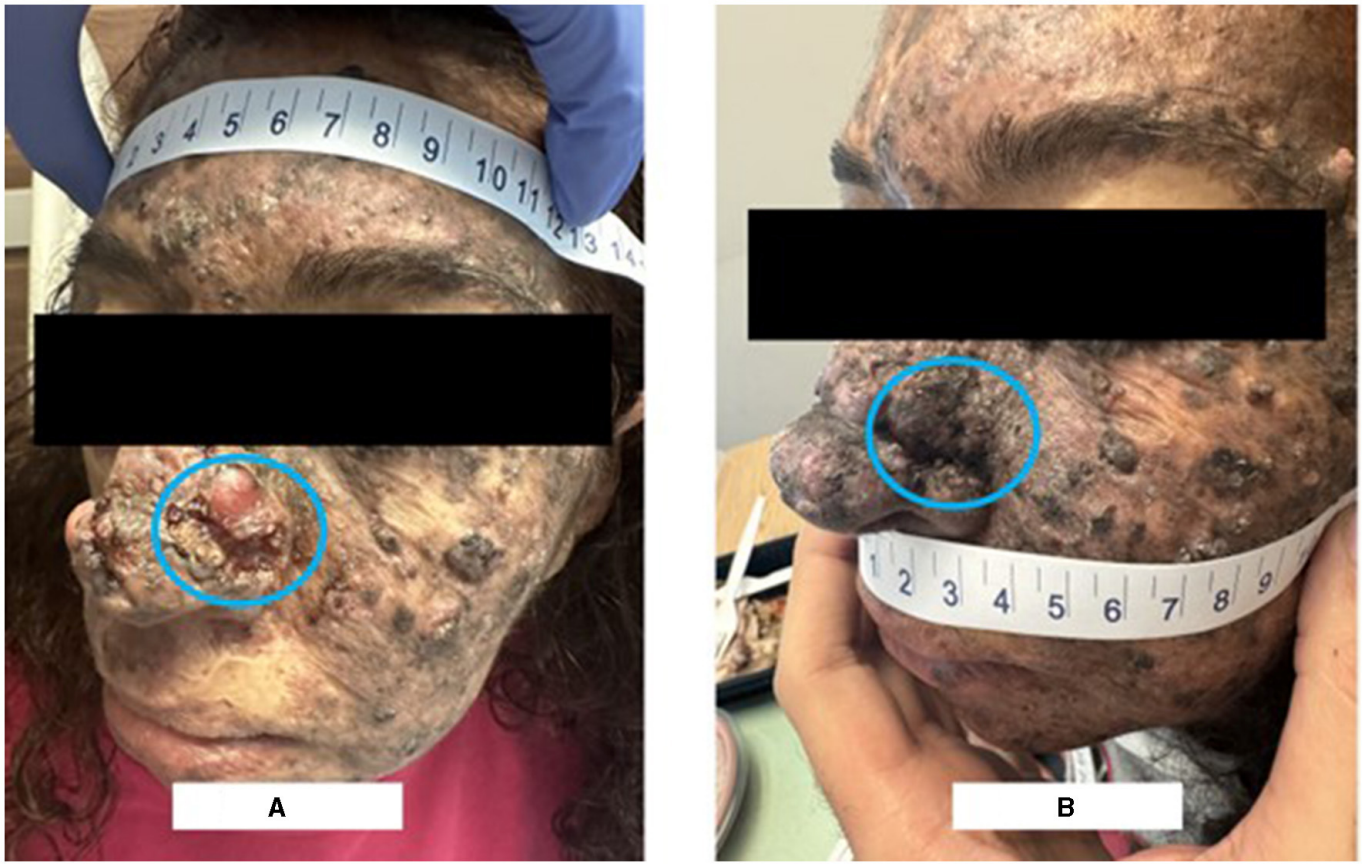
Clinical picture on presentation vs after 2 cycles of neoadjuvant pembrolizumab. **(A)** Shows the ulcerating mass over the left side of the nose. **(B)** Shows the disappearance of the left nasal lesion after 2 cycles of pembrolizumab.

A 49-year-old female, who is known to have Xeroderma Pigmentosum with no prior history of cancers, presented in August 2023 for evaluation and management of a growing ulcerating mass over the left side of the nose which was causing left-sided nasal obstruction (Figure 1A). Biopsy revealed moderately differentiated squamous cell carcinoma. On exam, the patient had an ulcerating 3 × 2 cm mass over the left nasal wall.

**Figure 2:**
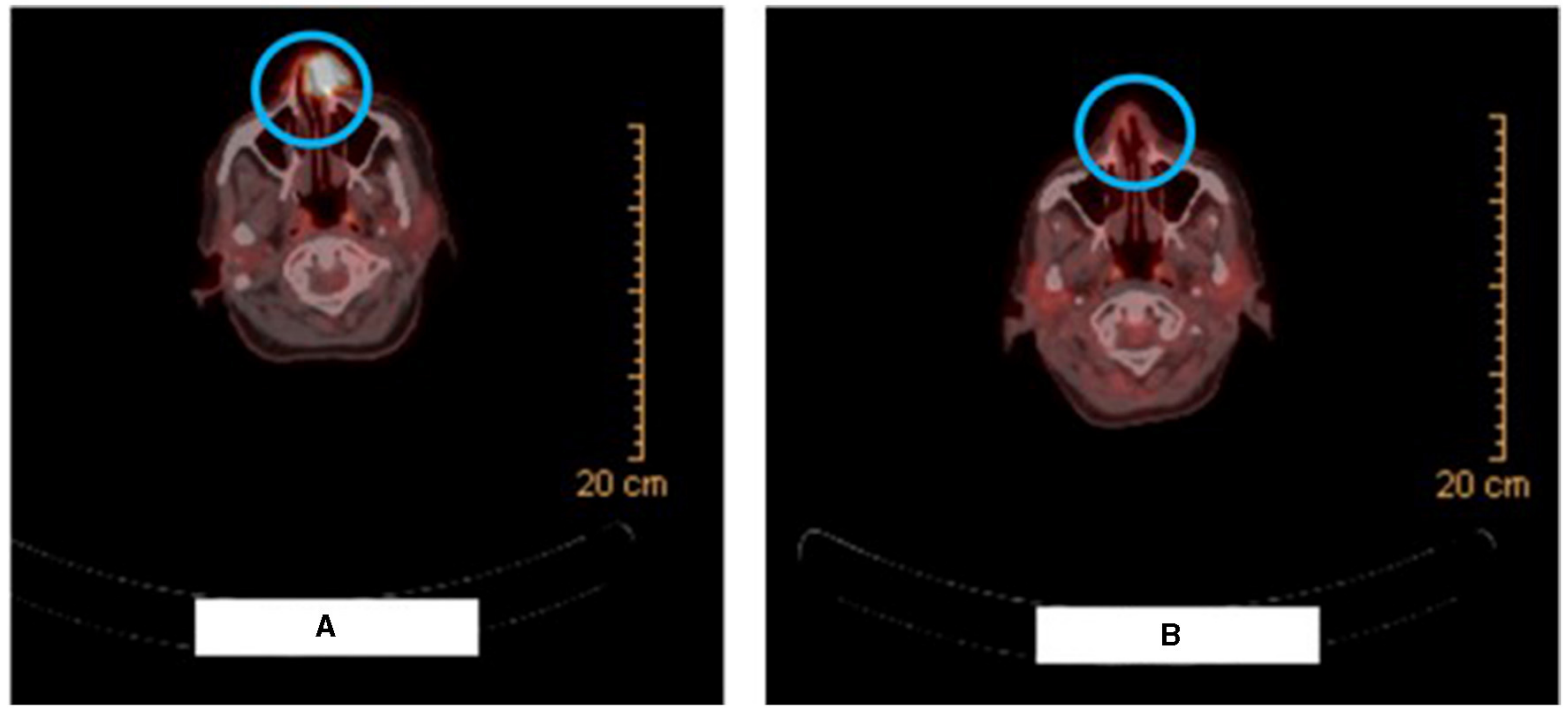
Radiologic picture on presentation vs after 2 cycles of neoadjuvant pembrolizumab. **(A)** Shows the PET-CT scan image demonstrating the radiotracer avid ulcerating soft tissue mass involving the left side of the nose causing nasal obstruction. **(B)** Shows the PET-CT scan image demonstrating resolution of the large radiotracer avid ulcerating soft tissue mass involving the left side of the nose and an interval decrease in activity, number, and size of the bilateral cervical lymph nodes.

PET-CT scan for the whole body with FDG was then performed (Figure 2A) and revealed a radiotracer avid ulcerating soft tissue mass involving the left side of the nose causing nasal obstruction, measuring 2.7 × 2.3 cm with SUV max of 16.2, representing the primary tumor. In addition, multiple radiotracer avid cervical lymph nodes were found, mainly submandibular, retromandibular, submental, jugulo-carotid, and mid and lower jugular lymph nodes. For example, a left submandibular lymph node found measured 1.2 × 0.7 cm with SUV max 5.7 and a right lower jugular lymph node found measured 0.8 × 0.7 cm with SUV max 4.5.

The patient's disease was considered as locally advanced, yet potentially resectable, nasal cSCC, stage IVA (T2N2cM0) according to the American Joint Committee on Cancer (AJCC), eighth edition. Her case was discussed at the multidisciplinary tumor board. Two treatment approaches were discussed: therapeutic intent dual immunotherapy with ipilimumab and nivolumab, considering her stage IVA disease, vs. surgical intent approach, considering her potentially resectable disease according to the locally advanced lymph nodes involvement and her young age coupled with her excellent performance status. The patient was adamant that she becomes disease-free. With her locally advanced disease, upfront surgery was not feasible. In respect to her wishes, the medical team had to explore other options and decided to resort to immunotherapy as a neoadjuvant treatment after which surgery can be rediscussed with the patient following 2 cycles of immunotherapy. As such, the patient was planned to receive 200 mg IV of the humanized monoclonal anti-PD1 antibody, Pembrolizumab, every 3 weeks for 2 years as well as radiation therapy.

PET-CT scan and MRI of the face were performed after 2 cycles of pembrolizumab. The PET-CT scan (Figure 2B) demonstrated resolution of the large radiotracer avid ulcerating soft tissue mass involving the left side of the nose and an interval decrease in activity, number, and size of the bilateral cervical lymph nodes. Similarly, the MRI showed a significant decrease in the size of the ulcerating mass in the left side of the nose and detected only a few prominent level IIa submandibular and submental lymph nodes (for example, a left level IIa lymph node measuring 1 × 0.8 cm). Furthermore, physical examination revealed the disappearance of the left nasal lesion which was previously present at the time of presentation (Figure 1B). The case was discussed at our multidisciplinary tumor boards discussion. Taking into consideration the significant morbidity that would arise from neck lymph node dissection, coupled with the complete clinical response of the cutaneous lesion itself, decision was to perform radiation therapy after an additional (third) cycle of pembrolizumab. She received a total of 66 Gray in 33 fractions and continued adjuvant pembrolizumab following the radiation therapy course with no adverse events.

## Discussion

In this report, we discussed the case of a 48-year-old female with xeroderma pigmentosum (XP) presenting with locally advanced potentially resectable nasal SCC. The tumor's location and size, coupled with its locally advanced lymph nodes involvement, would have necessitated a traumatic and challenging surgery with expected disfigurement post-operatively. At first, the medical team were reluctant to start the patient on pembrolizumab monotherapy due to limited evidence on its efficacy in resectable cSCC-HN and its limited evidence on XP patients. But, after thorough discussion with the patient and her family, and after a multidisciplinary discussion, the team proceeded with neoadjuvant intent pembrolizumab. Interestingly, this led to complete remission without the need for surgery.

ICIs efficacy in advanced and metastatic cSCC was investigated in several multicohort studies and clinical trials (15), the two main PD-1 inhibitors studied were cemiplimab (16–23) and pembrolizumab (24, 25).

The efficacy of cemiplimab in cSCC was studied in two phases. Initially, a phase I multicohort study involved 26 patients (20, 21), followed by a larger phase II EMPOWER-CSCC 1 trial with 193 patients (16–19, 22). Both studies had an open-label, multicenter design. The phase I trial demonstrated that cemiplimab was safe and effective, showing a 50% response rate [95% CI (30, 70)]. Among the 13 responding patients, 7 had responses lasting more than 6 months (20). These results were consistent in the phase II trial (18), where cemiplimab was given at different doses across three groups, with median times to response of 1.9 months for groups 1 and 2, and 2.1 months for group 3. About one-third of the patients had prior systemic treatments, most had surgery, and many had radiotherapy. In the metastatic cSCC cohort, 22 out of 29 responders had a duration of response (DOR) of 12 months or more, and in the locally advanced cSCC cohort, 12 out of 34 responders had a DOR of 12 months or more.

Pembrolizumab was evaluated in two phase II trials for cSCC: KEYNOTE 629 with 105 patients (24) and the CARSKIN trial with 57 patients in the expansion cohort (25). Both trials were open-label, single-arm, and multicenter. In KEYNOTE 629, the objective response rate was 34%, with complete responses in 4% and partial responses in 31% of patients. Among the 36 patients with a confirmed response, approximately 69% experienced durable responses longer than 6 months. With a median follow-up of about 10 months, the median progression-free survival (PFS) was 7 months, and the 1-year overall survival (OS) was 60% (24). Finally, in the CARSKIN trial, which only included treatment-naive patients, the objective response rate was 42%, with complete responses in 7% and partial responses in 35% of patients. In the expansion cohort, the response rate was higher in patients with PD-L1-positive disease (55%) compared to those with PD-L1-negative disease (17%), with a significant difference (*P* = 0.02) (25). After a median follow-up of 22.4 months in the primary cohort, the median PFS was 7 months, and the median OS was 25 months.

Thus, this led to the FDA approval of cemiplimab and pembrolizumab for the treatment of advanced and metastatic cSCC. We could not perform immunohistochemistry test for PD-L1 due to financial restrains, but this did not hinder initiation of immunotherapy as the benefit from pembrolizumab in cSCC was observed regardless of PD-L1 combined positive score (CPS) (24). However, a gray zone remains about the role of ICIs in resectable cSCC. For long, the mainstay of treatment of resectable cSCC-HN is surgical resection and adjuvant radiation therapy.

Currently, clinical trials are being conducted on the efficacy of ICIs in resectable cSCC. For instance, a phase II trial of 20 patients with resectable stage III/IV cSCC of the head and neck showed that 85% of participants achieved a pathologic response on cemiplimab, with 55% achieving a complete pathologic response, 20% achieving a major pathologic response and 10% achieving a partial pathologic response (26). Additionally, the 12 month disease-specific survival (DSS), disease-free survival (DFS), and OS are 95% [95% CI, (85.9, 100)], 89.5% [95% CI, (76.7, 100)], and 95% [95% CI, (85.9, 100)], respectively (27). Another cohort study of 27 patients with resectable cSCC (mainly in the head and neck region) treated with neoadjuvant cemiplimab or pembrolizumab showed an overall pathologic response rate of 47.4% and the overall radiologic response rate was 50.0%. Further, the 1-year recurrence-free survival rate, progession free survival, DSS, and OS were 90.9% (95% CI, 50.8%−98.7%), 83.3% (95% CI, 27.3%−97.5%), 91.7% (95% CI, 53.9%−98.8%), and 84.6% (95% CI, 51.2%−95.9%) respectively (28). However, further research is needed to establish the benefits of ICIs monotherapy in cSCC-HN.

In addition to immunotherapy, our decision to use radiation therapy for this patient was based on two main factors: preventing tumor recurrence and reducing the incidence of skin cancers in the treated area, as studies have shown radiation therapy to be effective for XP patients (29).

Deinlein et al. were the first to report the efficacy of pembrolizumab among patients with XP-associated cSCC with rapid response observed after only three cycles (30). A few other reports also suggested the safety and effectiveness of immunotherapy and radiation therapy for metastatic cSCC (30–32). However, there are no clinical studies or randomized-control trials to date that confirm the safety and efficacy of immunotherapy and radiation among patients with XP. With high predisposition to skin cancers and immunogenicity based on high tumor mutation burden, this patient population is actually a suitable target for future studies on the effectiveness of immunotherapy. Our patient serves as a good model for the study of efficacy of immunotherapy and radiation on skin cancers in XP patients with cSCC.

To our knowledge, this is the first documented case outside of a clinical trial of a patient with XP with locally resectable cSCC-HN who was treated with pembrolizumab monotherapy. This regimen led to a complete response of the tumor and thus spared the patient from any surgical intervention and disfigurement. Future follow-up is needed to document potential recurrence.

In light of early clinical trials and this case report, it is crucial to acknowledge that surgery is not the only option for resectable cSCC-HN and thus we should consider the option of neoadjuvant ICIs for patients reluctant on undergoing surgery, because this might lead to complete response. Phase III trials are needed to compare ICI monotherapy to the standard of care. Also, clinical trials are needed to investigate the potential role of prophylactic ICIs in patients with XP who are at higher risk for cSCC.

## Data Availability

The original contributions presented in the study are included in the article/supplementary material, further inquiries can be directed to the corresponding author.
